# Report of State Board of Medical Examiners

**Published:** 1895-03

**Authors:** William C. Wey, Maurice J. Lewi

**Affiliations:** President; Secretary’s Office, 78 West 82d Street, New York; Secretary; Secretary’s Office, 78 West 82d Street, New York


### ANNUAL REPORT OF THE STATE BOARD OF MEDICAL EXAMINERS, REPRESENTING THE MEDICAL SOCIETY OF THE STATE OF NEW YORK.

SECRETARY'S OFFICE, 78 WEST 82D STREET,
NEW YORK, February 5, 1895.

*To the Medical Society of the State of New York :*

GENTLEMEN—During the past year the exemptions from the medical licensing law having nearly ceased, the law placed on the statute books largely through the energetic efforts of this society may be said to have been in normal action. The following statistics furnish a fair idea of our work during the past year, from January 1, 1894, to January 1, 1895.

(These statistics should not be confused with those prepared by the regents examination department, as their records are made up annually to conform with their general method of compilation, which is based on the opening and closing of the academic year, August 1, 1893, to July 31, 1894.)

Number of candidates actually examined, 536.

| | Examined. | Passed. | Per cent. Rejected. |
|---|---|---|---|
| Before the State Board of Medical Examiners, representing the Medical Society of the State of New York | 470 | 316 | 32.7 |
| Before the State Board of Medical Examiners, representing the Homeopathic State Medical Society | 59 | 46 | 22.0 |
| Before the State Board of Medical Examiners, representing the Eclectic Medical Society of the State of New York | 7 | 3 | 57.1 |

| | | |
|---|---|---|
| Number of candidates rejected a second time | | 17 |
| " " " three or more times | | 6 |
| " " " during the year who have been unable thus far to receive a license | | 54 |
| Number of defective registrations corrected | | 31 |
| By state board | 22 | |
| By homeopathic board | 3 | |
| By eclectic board | 6 | |

It was thought, in 1891, that the average number of candidates who would yearly appear before us for examination would be about 400. This estimate was based on the theory that the law admitting candidates to examinations was so stringent as to exclude all not well prepared to pass our tests. Candidates for admission must be of good moral character, as certified by two reputable practitioners of medicine; they must be graduated doctors of medicine who have attended at least three full courses of lectures at a registered medical school in three different years and must have met the preliminary education prescribed by law. These exactions shut out the incompetent and in consequence the percentage of rejections must be considered in this light. The discrepancy in the estimated and actual number of candidates is due to two causes. Whereas, prior to 1891 the number of physicians locating yearly in the state was from 700 to 800, (and this can only be estimated, as no exact figures are obtainable,) now there are fewer than 400. The applicants numbered 536, as above stated. The natural increase in population might account for a corresponding increase in the number of medical men; but the main cause is the fact that our state licenses have grown to be documents of such importance that most of the states having boards of medical examiners accept them as licenses to practise without exacting added medical tests.

The selection of questions properly grouped for examination purposes is a task to which much of our time has been devoted, and we invite criticism of our work in this particular with the hope of improving on a system which has been generally commended. It may be of interest to the society to know that the questions are originally framed by all of the examiners, each chair through its three representatives submitting, as occasion requires, 180 questions. A total of 1,080 questions thus obtained is referred to the question committee, composed of two members from each board. Here the questions are carefully studied and sifted, only such being accepted as are considered fair, that is, as requiring knowledge which every practitioner of medicine should have. To the editor of the committee is then referred all accepted questions, who in turn groups them in sets of fifteen in each topic, covering as nearly as possible every sub-branch of the subject under consideration. On requisition of the regents these groups are forwarded to Albany, and the regents become responsible for printing and for the language used in framing the questions for publication. Thus far twenty-one regular and three special examinations have been held, at which 2,520 questions have been asked or 360 questions in each topic. A duplication of questions thus propounded and published to the world is not desirable, but we have now reached a point where this is becoming essential if we desire to be freed from the charge of propounding queries which belong in the special rather than in the general field of medicine. Other similar bodies of examiners submit and publish questions which indicate a far more difficult test for license, but the method of marking must be comparatively lenient as judged by the percentage of rejections. We feel it our duty as examiners not to prevent a given percentage of those applying from being licensed in order to maintain a standard, preferring to act favorably on the application of all, no matter how great the number, whom we believe to be competent to practise medicine.

Under the provisions of the law we are permitted to indorse as sufficient for practice the licenses issued by other state boards of medical examiners, provided the standards of these bodies are not lower than ours. Numerous applications from various states have been received for this indorsement, but on investigation we have in each instance found that the standard was not sufficiently high to warrant us in approving the documents submitted. We earnestly appeal to the society to continue the work, inaugurated by the committee on legislation in 1892, of having bi-monthly transcripts of registration made from the books of the county clerks throughout the state. In this manner only can a comprehensive record of the registration throughout the state be secured, thus furnishing the opportunity of verifying or correcting the work of the county clerks. During the year many letters have been addressed to us calling attention to persons who are practising medicine in various parts of the state without legal authority. It has been our custom to investigate all such cases and to advise prosecution where the offender is a new-comer; but when the case is one which has existed before September 1, 1891, viz., before the passage of the law under which we were called into existence, we have advised against interference. The unfortunate dropping of the word misdemeanor from its former place in the work of codifying the medical laws as reported to the legislature by the revision committee two years ago, has robbed the statute of much of its worth. Through the energetic efforts of your committee on legislation both branches of the legislature last year unanimously passed a bill correcting this defect, but notwithstanding heroic efforts on the part of the profession throughout the state, the then governor vetoed the bill. In order to maintain the dignity of the licensing power now entrusted to our care, we earnestly urge that the committee on legislation be again instructed to introduce this measure in the present legislature in the hope of restoring the statute in its integrity.

Since our last meeting two adjoining states have established state boards of medical examiners, the laws appointing them being modeled after our own.

The preliminary education of medical students and candidates for the licensing degree in this state is not satisfactory. While the standard is far above that established in other states, it is still insufficient and not on a par with the requirements exacted from students in other professions in our state. The following was unanimously carried at a meeting of our board, with the intention that it be embodied in our report to your honorable body :

*Resolved*, That, in the opinion of this board, the best interests of the public and of the medical profession would be materially advanced by gradually increasing the minimum requirements as to general preliminary education, till no candidate be entitled to matriculate in 1897 at a degree granting medical school in this state who has not completed at least a full high school course.

Respectfully submitted.

WILLIAM C. WEY, M. D.,

*President.*

MAURICE J. LEWI, M. D.,

*Secretary.*

